# Influences of glyphosate residues and different concentrate feed proportions in dairy cow rations during early gestation on performance, blood parameters, functional properties and DNA damage of blood cells in cows and their offspring

**DOI:** 10.1371/journal.pone.0286995

**Published:** 2023-06-09

**Authors:** Ann-Katrin Heymann, Karina Schnabel, Fabian Billenkamp, Susanne Bühler, Jana Frahm, Susanne Kersten, Dirk von Soosten, Ulrich Meyer, Sven Dänicke

**Affiliations:** Institute of Animal Nutrition, Federal Research Institute for Animal Health, Friedrich-Loeffler-Institut, Braunschweig, Germany; Tokat Gaziosmanpasa Universitesi, TURKEY

## Abstract

Maternal exposure to various stimuli can influence pre- and postnatal development of the offspring. This potential has been discussed for glyphosate (GLY), active substance in some non-selective herbicides. Accordingly, present study investigated putative effects of GLY residues in rations on cows and their offspring. Dams received either GLY-contaminated (GLY groups) or control (CON groups) rations combined with low (LC groups) or high (HC groups) concentrate feed proportions (CFP) for 16 weeks during mid- and late lactation and early gestation (59±4 days at beginning of GLY exposure; mean±SE). During this feeding trial, average daily GLY exposures of dams were 1.2 (CON_LC_), 1.1 (CON_HC_), 112.5 (GLY_LC_) and 130.3 (GLY_HC_) μg/kg body weight/d. After a depletion period (107±4 days; mean±SE) and calving, blood samples of dams and their calves were collected (5–345 min after birth) before calves were fed colostrum and analyzed for hematological and clinical-chemical traits, redox parameters, functional properties of leukocytes and DNA damage in leukocytes. No evidence for malformations of newborn calves could be collected. At parturition, most analyzed blood parameters were not affected by dietary treatment of dams during gestation. Significant GLY effects were observed for some traits, e.g. blood non-esterified fatty acids (NEFA) in calves. These deviations of GLY groups from CON groups likely resulted from strong time-dependent responses of NEFA levels within the first 105 minutes after birth and before colostrum intake (Spearman´s rank correlation R = 0.76, p<0.001). Additionally, significant GLY effects did not result in differences in measures that were beyond normally observed ranges questioning a pathological relevance. In summary, no evidence for teratogenic or other clear effects of GLY or CFP on analyzed parameters of dams and their newborn calves could be collected under applied conditions. However, detailed studies including GLY exposure during late and complete gestation period would be needed to rule out teratogenic effects.

## Introduction

Health and development of the fetus and calf depend on successful management and nutrition of the cow [1]. Additionally, maternal and, therefore, fetal exposure to various stressors, such as environmental conditions, can adversely affect fetal development [2]. Possible stressors include environmental conditions, such as high temperatures, dietary deficiencies, toxicities, and xenobiotics such as glyphosate-based herbicides (GBH) [2]. Glyphosate (GLY) is used as an active substance in non-selective herbicides worldwide [3]. Although some countries are going to restrict the usage of GBH (e.g. Germany [4]) due to growing public concerns and controversies in literature about possible adverse GLY effects on human and animal health, most countries continue to allow the use of GBH. In Europe, GLY residues can be found in human food [5] and in livestock feed produced under agricultural conditions using formerly legal GLY administration [6] or imported soybean meal [7]. This consequently indicates a regular oral exposure of humans and animals and, therefore, possible *in utero* exposure of developing fetuses. No GLY residues were detected in dairy cow [6] and human milk [8]. Nevertheless, effects of *in utero* GLY exposure on offspring and orally exposed dams are of scientific interest [9–12]. In human pregnancies, a correlation between GLY exposure and pre-term birth as well as increased numbers of spontaneous abortions were reported in women of an Ontario farm population [13]. Malformations in piglets observed in a field investigation were associated with 0.87 to 1.13 mg GLY/kg contamination in sow feed [14]. In contrast, 0.08 to 1.52 mg GLY/kg dry matter gestation diet did not influence frequencies of stillborn piglets or kinky tails [15]. In laboratory animals, such as Wistar rats, contradictory results were reported. The offspring of rats treated with 70 mg GLY/kg body weight (BW)/d from gestational day 5 until postnatal days 15 or 60 did not show any malformations [9]. In contrast, dose-dependent proportions of skeletal alterations were observed for 57.3% of offspring maternally treated with 1000 mg GLY-solution/kg on gestation days 6 to 15. In control animals, 15.4% showed these alterations [16]. Absence of GLY effects was reported for offspring’s birth weight in studies with rats treated with doses varying from 50 to 1000 mg GLY/kg BW/d during different periods of gestation [9, 10, 16]. Additionally, possible GLY effects on offspring’s performance and general development, influences on blood parameters such as clinical-chemical traits were investigated [17]. Serum triglyceride levels were increased in mice offspring on gestational day 19 after maternal exposure of 35 mg GLY/d [17].

To our knowledge, there are no *in vivo* trials examining GLY effects on dairy calves, while studies on laboratory animals were reported [9, 10, 16]. Consequently, we hypothesized that upon common agricultural conditions *in utero* GLY exposure in early gestation might also affect health and metabolism of the developing fetus as well as the neonatal calf. Secondly, we considered GLY effects may be differently pronounced in the calves of dams fed high or low concentrate feed proportions (CFP). Other secondary hypotheses explored included an evaluation of effects of GLY exposure and CFP on metabolites in the dams following GLY exposure, a depletion period and parturition.

Pregnant cows investigated in the present study originated from a feeding trial under common practical conditions in agriculture as recently described by different authors [6, 18–20]. That trial indicated that 16 weeks of GLY exposure (average GLY exposure: 122.7 μg/kg BW/d GLY) did not lead to adverse effects on performance [6], serum metabolites [6, 19], ruminal microbiome [18], liver health [19], hematology, functional properties of immune cells, oxidative status, and DNA integrity of blood cells in dairy cows in mid-lactation [20]. Different CFP affected most of analyzed parameters [6, 18–20]. Feeding different CFP as common agricultural practice resulted in different fiber content, energy and nutrient supply, and affected consequently ruminal milieus. This feeding strategy was used, since GLY effects on ruminal microbiota were reported in dependency of fiber content *in vitro* [21]. The present study aimed to analyze putative long-term effects of dietary GLY exposure during mid- and late lactation and early gestation on dams and their offspring and, thus, filling an existing lack of knowledge. Hereby, the parturition (before calves had colostrum intake) represented the earliest time point for assessing possible effects of maternal nutrition on newborn calves.

## Materials and methods

The animal trial for the present study was conducted at the experimental station of the Institute of Animal Nutrition, Friedrich-Loeffler-Institut (FLI), in Braunschweig, Germany in accordance with the German Animal Welfare Act and was approved by the Lower Saxony State Office for Consumer Protection and Food Safety (LAVES, 33.19-42502-04-15/1858).

### Experimental design and feeding

Pregnant cows used in this study originated from a feeding trial described in detail elsewhere [6, 20]. In a two by two factorial design, dairy cows were fed rations, which had different CFP and GLY-treated or non-treated feedstuffs, over a period of 16 weeks in mid- and late lactation. Days in milk of pregnant cows amounted to 161 ± 5 (mean ± SE) at the beginning of the study, while varying from 82 to 213 days. In short, experimental feedstuff was grown on FLI Research Station. Parts of wheat and peas were treated with Roundup Record® containing 720 g glyphosate/kg solution (007525–60/MOT), Monsanto, Agrar Deutschland GmbH (Düsseldorf, Germany) in pre-harvest application, while the other parts as well as maize and grass remained untreated [6]. In order to avoid cross-contaminations, GLY-treated and non-treated feedstuffs were separately harvested and stored, and additionally homogenized in the process of feed manufacturing. The feeding trial was conducted with four feeding groups arranged in a complete two by two factorial design and represented a worst-case scenario for common realistic agricultural conditions in Germany 2014 according to the legislation at that time. Cows were blocked by number of lactations, mean BW, daily feed intake and fat corrected milk prior to the trial and randomly allocated to one of four experimental groups as described [6, 20]. The aim was to analyze interactions between CFP and GLY residues in the cows’ diets. Cows were fed according to nutritional needs as defined in [22] and as described in detail in [6]. For this purpose, high (HC groups) or low CFP (LC groups) in the diets were fed ad libitum as a total mixed ration with (GLY groups) or without GLY contaminations (CON groups) resulting in the four experimental groups (CON_HC_, CON_LC_, GLY_HC_, GLY_LC_). In LC groups, diets consisted of 21% maize silage, 42% grass silage, 7% straw and 30% concentrate, while for HC groups diets contained 11% maize silage, 22% grass silage, 7% straw and 60% concentrate based on dry matter [6]. Detailed descriptions of chemical compositions and nutritional analyses of individual feedstuffs can be found in [6].

At the beginning of the 16-week feeding trial, 39 cows had been pregnant for 59 ± 4 days (mean ± SE) (CON_HC_, n = 9; CON_LC_, n = 11; GLY_HC_, n = 10; GLY_LC_, n = 9). After terminating the feeding of experimental diets, pregnant cows were further observed until parturition. During this depletion time (defined as time span between termination of feeding the experimental diets to the dams until the following parturition), which ranged from 58 to 158 days (107 ± 4 days mean ± SE), cows received diets untreated with GLY consisting of grass silage and maize silage as well as CFP in accordance with their individual milk yield. After parturition, dams and their calves were examined and blood samples taken before calves were fed colostrum.

### Examination of performances of calves

Calving ease was evaluated on a scale of 1 to 3. Scores indicated that no (1), little (2) or much assistance, including technical equipment (3), was needed at parturition. The BW of the calves was recorded within the first 24 h after birth, on the 21^st^ and 42^nd^ day of life. Stillborn calves were excluded from further analysis, but underwent pathology and histopathology by the University of Veterinary Medicine Hannover, Foundation (TiHo), Hanover, Germany. Within the first three days of life, heart rate, body temperature, and respiratory frequency were measured. Additionally, on the same day, calves were examined for evidence of anatomical malformation or physiological misfunction consistent with teratogenic effects of GLY exposure.

### Blood sampling and serum metabolites

Blood samples of unsuckled calves and their dams were collected within 5–345 min (70 ± 92 min; mean ± SD) after calving from a *Vena jugularis externa* using serum tubes and tubes containing sodium-heparin or ethylenediaminetetraacetic (EDTA). Serum activities of alkaline phosphatase (AP), alanine aminotransferase (ALT), aspartate aminotransferase (AST), glutamate dehydrogenase (GLDH), γ-glutamyltransferase (GGT) and concentrations of total bilirubin, total protein, albumin, cholesterol, triglycerides, non-esterified fatty acids (NEFA), glucose, β-hydroxybutyrate (BHB), urea, uric acid, phosphorus, chloride, potassium and sodium were measured (Eurolyser®, Type VET CCA, Eurolyser Diagnostica GmbH, Salzburg, Austria).

### Hematological evaluation

EDTA-blood samples of calves and dams were analyzed for total and differential blood cell profiles using automatic analyzer (Celltac-α, MEK-6450, Nihon Kohden, Tokyo, Japan) for leukogram, erythrogram, and platelets.

### Antioxidative status

Determination of total superoxide dismutase (SOD) and glutathione peroxidase (GPx) activities was conducted in duplicates with EDTA-blood samples using Ransod superoxide dismutase- and Ransel glutathione peroxidase assays (Randox Laboratories, Crumlin, UK) according to the manufacturer’s protocol and adjusted as described in [23]. For SOD the inter- and intra-assay coefficients of variation (CV) were 14.43% (inter) and 11.35% (intra). The inter- and intra- assay test performances for GPx revealed CVs of 8.64% (inter) and 5.04% (intra), respectively. For normalization of the enzyme data, hemoglobin concentration of erythrocyte lysate was measured using an automatic analyzer (Celltac-α, MEK-6450, Nihon Kohden, Tokyo, Japan).

For measurement of ferric reducing ability of plasma (FRAP), the anti-oxidative capacity was measured according to [24]. Antioxidative reactions in plasma were photometrically measured for 15 min at 593 nm (Tecan infinite M 200, Grödig, Austria).

### RNA isolation and quantitative real-time PCR

For gene expression analysis, RNA was isolated from 2 mL EDTA blood samples. For erythrocyte lysis, 10 mL ddH_2_O with additional 1080 μL 8.8% NaCl added after 15 sec were used for sample of dams. For samples of calves, 48 mL lysis buffer containing 150 mM NH_4_Cl, 10 mM KHCO_3_, 0.1 mM EDTA (pH 7.47) was used instead of ddH_2_O followed by an incubation (10 min, 4°C). Pellets were resuspended twice in 1 mL ddH_2_O with additional 108 μL 8.8% NaCl after 15 sec or for samples of calves, pellets were dissolved in 2 mL lysis buffer and incubated on ice for 5 min. Total RNA was isolated using the kit NucleoSpin® RNA (Macherey-Nagel GmbH & Co. KG, Düren, Germany) according to the manufacturer’s protocol and dissolved in RNAse-free ddH_2_O.

Ten genes of interest with relation to blood parameters that displayed significant changes upon experimental conditions were subjected to quantitative real-time polymerase chain reaction (qRT-PCR). Here, 750 ng RNA isolated from the EDTA blood samples of calves and their dams were used to synthesize cDNA by the qScript™ cDNA Synthesis Kit (Quanta Biosciences™, Inc, Gaithersburg, MD, USA) according to the manufacturer’s protocol. Gene-specific primer pairs were designed using Primer-Blast [25], Beacon Designer (Free Edition Premier biosoft) as well as Primer3 version 4.0.0 [26, 27] and used for qRT-PCR as described previously [28]. Expression and values of quantification cycle (Cq) of genes were obtained by CFX Maestro™ 1.1 (Bio-Rad Laboratories, Inc, Hercules, CA, USA) using regression mode for Cq determination. Information about selected primers is shown in S1 Table. Normalized expression of genes of interest was calculated by using reference gene expression of *RPS9* (ribosomal protein S9), *UCHL5* (ubiquitin c-terminal hydrolase L5) and *UXT* (ubiquitously expressed prefoldin like chaperone) as normalization factors and taking primer specific efficiencies into consideration. Prior to statistical analyses, gene expression data was subjected to logarithmic transformation and transformed back after analyses for better interpretation.

### Flow cytometric analyses

Functional parameters (T-cell phenotyping, intracellular reactive oxygen species (ROS) production, phagocytosis, apoptosis) for polymorphonuclear leukocytes (PMN) and peripheral blood mononuclear cells (PBMC) were analyzed in EDTA whole blood samples using flow cytometry (FACS Canto II, BD Biosciences, San Jose, CA, USA). Respective cells were defined and gated according to their size and granularity based on forward and side scatter measurements. Evaluation of at least 10,000 cells was conducted with FACS Diva software 6.1.3. (BD Biosciences, San Jose, CA, USA) in a standardized manner. Unless otherwise stated, results were expressed as proportions or mean fluorescence intensities (MFI). All methods used in the present study are described below and in [20].

### T-cell phenotyping

For phenotyping of lymphocytes, EDTA blood samples were double-stained with monoclonal antibodies for CD4+ (mouse anti-bovine CD4+:FITC (fluorescein isothiocyanate); Bio-Rad, Hercules, CA, USA) and CD8+ (mouse anti-bovine CD8+:PE; Bio-Rad Hercules, CA, USA) or their related isotype controls (mouse IgG2a negative control RPE, and mouse IgG2b: FITC negative control; Bio-Rad, Hercules, CA, USA). Further procedure was conducted as described above and as described in [20].

### Intracellular production of reactive oxygen species

The ability of PMN to produce intracellular ROS was measured by flow cytometry based on oxidation of non-fluorescent dihydrorhodamine 123 (DHR) to the fluorescent metabolite rhodamine 123 (R123+) by free radicals. EDTA whole blood samples were incubated with 40 μM DHR (Molecular Probes, Eugene, OR, USA) alone (basal/unstimulated) or with additional 30 μM TPA (12-O-tetradecanoylphorbol-13-acetate; Sigma-Aldrich, Taufkirchen, Germany) to induce an oxidative burst (stimulated) and further processed as described [20]. Results of flow cytometric measurements were expressed as the percentage of R123+ PMN and their MFI, representing the conversion of DHR per cell.

### Phagocytosis

The Phagotest^TM^ kit (Glycotope Biotechnology GmbH, Heidelberg, Germany) was used for determination of phagocytic activity of PMN according to the manufacturer´s protocol. In brief, heparinized blood samples were incubated with FITC-labelled *E*. *coli*, quenched, washed and stained with propidium iodide (PI). Flow cytometric measurements (FACS Canto II, BD Biosciences, San Jose, CA, USA) of capacity and proportions of cells ingesting bacteria were conducted in duplicates within 60 min after staining.

### Apoptosis

Determination of apoptotic PMBC and PMN cells in EDTA whole blood samples was performed using the FITC Annexin V-Apoptosis Detection Kit II (BD PharmingenTM, BD Biosciences, San Diego, CA, USA) according to the manufacturer´s protocol and as described earlier [20]. In brief, after lysis of erythrocytes by hypotonic shock, blood samples were incubated with and without PI and FITC-coupled annexin for 15 min in the dark and analyzed in duplicates by flow cytometry within one hour. Different stages of apoptosis were distinguished: doubled-stained cells (FITC Annexin V positive, PI positive) marked late apoptotic cells, while single-stained cells were identified as early apoptotic cells (FITC Annexin V positive, PI negative) or necrotic cells (FITC Annexin V negative, PI positive).

### DNA damage indicators

To detect DNA damages in individual bovine leukocytes, the gel electrophoresis based comet-assay was conducted as described [29] with some modifications [20]. In short, EDTA blood samples mixed with 0.5% low melting point agarose (NuSieve Ag, Lonza, Basel, Switzerland) was layered on glass slides precoated with a 1.5% medium electroendoosmosis (MEEO) agarose base layer (Karl Roth, Karlsruhe, Germany). After incubation on ice, erythrocyte lysis, electrophoresis in alkaline electrophoresis buffer, neutralization and drying of slides, DNA was stained with DAPI solution (Roti-Mount FluorCare, Carl Roth GmbH, Karlsruhe, Germany). At least 100 nuclei per slide in triplicates per sample were documented by fluorescence microscopy (Leica DMI 6000b, Leica Microsystems CMS GmbH, Wetzlar, Germany) and the level of DNA damage of each single nucleus were evaluated using CASPlab software [30]. Percentage of DNA in the tail in relation to total DNA content as well as the Olive tail moment, as product of DNA content in the tail [%] and the tail length [31] served as indicators for DNA damage [32].

### Calculations and statistical analyses

For statistical analyses of blood parameters, 38 calves were included, while one calf (CON_LC_), which had died within 24 h after birth, was excluded from analysis of average daily gain, heart rate, respiratory rate, and body temperature. Average daily gain of calves was calculated by dividing differences between BW at day 42, day 21 and after birth by corresponding timespans.

Unless otherwise stated, measured parameters were analyzed using MIXED procedure in SAS (v9.4). As the design is 2x2 factorial, treatment (GLY; GLY or CON diet) and CFP (HC or LC diet) of dams as well as their interactions (GLY*CFP) were set as fixed factors. Depending on distribution of residuals (proc univariate, Shapiro-Wilk test), statistical analyses were conducted on untransformed or transformed (proc transreg or proc rank) data. If Box-Cox transformation was conducted, respective optimal λ was used as exponent. For rank analyses, anovaf option as well as repeated statement with an unstructured covariance model (un(1)) were added for F-test computing and mapping heterogenous variances. Data was presented as Lsmeans (untransformed, Box-Cox back-transformed or log back-transformed for gene expression data) or as means and respective 95% confidence limits depending on method. Multiple comparisons of means were conducted with Tukey-adjusted p-values (proc diff). Considering that data of many parameters were not normally distributed, Spearman´s rank correlations were calculated in RStudio version 1.2.5042 [33], R version 4.0.3 [34]. Results were declared as significant when p≤0.05. Figures were created by using R packages cowplot [35], ggplot2 [36], ggpubr [37], gridExtra [38], gtable [39], openxlsx [40], readxl [41] in RStudio version 1.2.5042 [33], R 4.0.3 [34].

## Results

### Performance of cows and calves

Performance of all cows (BW, milk yield, dry matter intake) during the main feeding experiment are presented in detail elsewhere [6, 20]. Taking only pregnant cows into consideration (n = 39), average daily GLY exposures of dams were 1.2 (CON_LC_), 1.1 (CON_HC_), 112.6 (GLY_LC_) and 130.3 μg/kg body weight/d (GLY_HC_) during the feeding trial of 16 weeks in mid- and late lactation and during early gestation. The mean depletion period (time between termination of experimental feeding and parturition) amounted to 107±4 days (mean ± SE), while varying between 58 and 158 days. Gestation period of dams varied between 258 and 294 days and was not influenced by dietary treatment (GLY, CFP) of dams (Table 1).

**Table 1:** Effects of glyphosate residues and different concentrate feed proportions in diets fed for 16 weeks in mid- and late-lactation during early gestation on gestation length of dairy cows and performance parameters of newborn calves.

	Group of dams				p-value		
	CON_HC_	CON_LC_	GLY_HC_	GLY_LC_	GLY	CFP	GLY*CFP
**Dams**	n = 9	n = 11	n = 10	n = 9			
Gestation time [d]^$^	279 _[274, 283]_	282 _[279, 286]_	279 _[273, 285]_	280 _[277, 283]_	0.594	0.195	0.141
Calving ease	1.7 _[__1__,__2__,__3__]_	1.3 _[__1__,_ _1__,__6__]_	1.3 _[__1__,__1__,__6__]_	1.7 _[__1__,_ _2__,__3__]_			
**Calves**	n = 9	n = 11	n = 9	n = 9			
Sex ratio (n = male/female)	6/3	4/7	5/4	4/5			
Birth weight [kg]^§^	45.4 _[41.3, 49.6]_	42.7 _[38.7, 46.7]_	45.7 _[41.5, 49.9]_	43.4 _[39.2, 47.6]_	0.812	0.222	0.910
Respiratory rate [breaths/min]^§^	55 _[__46__, 65]_	57 _[__48__, 65]_	49 _[__39__,_ _58__]_	49 _[__39__,_ _58__]_	0.135	0.881	0.900
Heart rate [beats/min]^§^	139 _[127, 151]_	133 _[122, 144]_	145 _[133, 157]_	130 _[118, 142]_	0.752	0.083	0.428
Body temperature [C°]^§^	38.6 _[38.4, 38.8]_	38.5 _[38.4, 38.7]_	38.5 _[38.3, 38.7]_	38.6 _[38.4, 38.7]_	0.642	0.883	0.525
Average daily gain [g/d]^+^	823 _[748, 897]_	828 _[757, 899]_	819 _[745, 894]_	760 _[685, 834]_	0.328	0.459	0.376

Values are presented as non-transformed Lsmeans or means ($) with 95% confidence limits [lower limit, upper limit]. Calves stillborn at parturition (n = 3) are excluded for all parameters, while data of one calf (CON_LC_) which had died days after parturition was only excluded for performance parameters (§). Calving ease was evaluated on a scale of 1 to 3. Scores indicated that no (1), little (2) or much assistance including technical equipment (3) was needed at parturition (+) average daily gain until day 42 after birth. CFP = concentrate feed proportions; CON = control; GLY = glyphosate; HC = high concentrate feed proportions; LC = low concentrate feed proportions.

Out of 41 calves, including two twin parturitions, 38 were clinically normal at parturition. Three calves were stillborn (two from twin births; 1x CON_LC_, 2x GLY_HC_), and one additional calf (CON_LC_) died within 24h of life. Pathological examination of stillborn calves revealed cardiovascular failure due to unclear reasons as cause of death. Statistical analyses did not indicate an influence of dietary treatments of dams during early gestation (GLY, CFP) on birth weight, respiratory rate, heart rate, and body temperature (p ≥ 0.083, Table 1) of the calves. Average daily gain was 0.8 kg/d in all groups corresponding to 78.2 kg average BW at day 42 of life and remained unaffected by treatments of dams in early gestation (p ≥ 0.328, Table 1).

### Blood parameters

#### Serum metabolites

**Fig 1:**
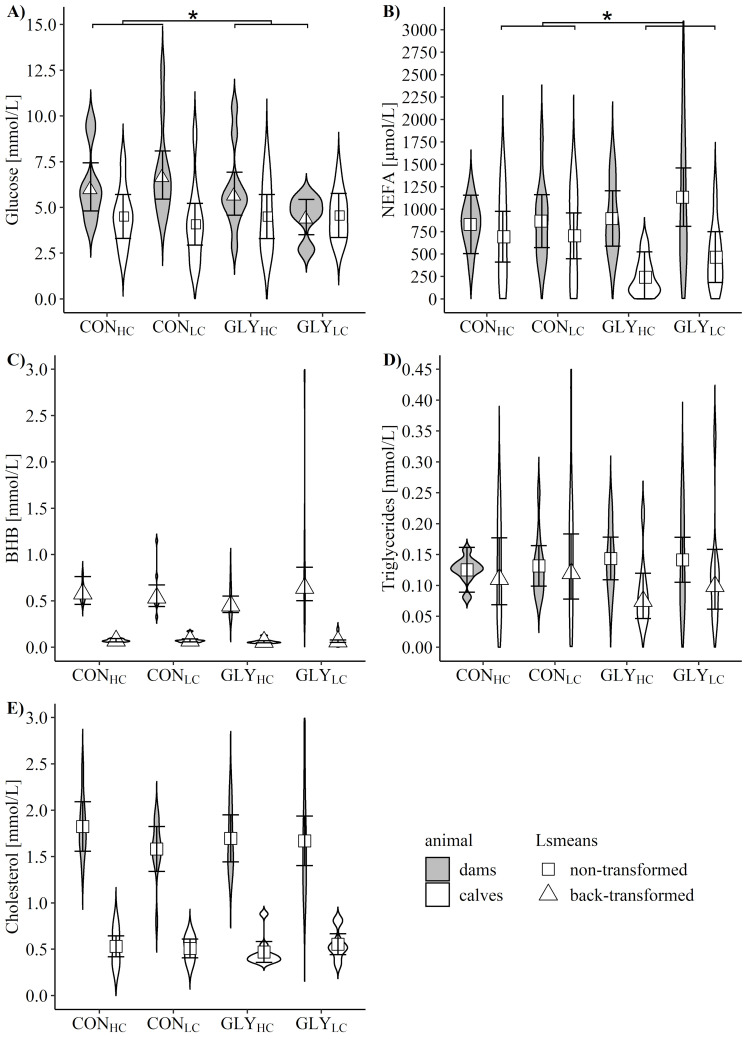
Influence of glyphosate residues and different concentrate feed proportions in diets fed during early gestation on serum metabolites in dams and calves after parturition. Depicted are serum glucose (A), non-esterified fatty acids (NEFA, B), β-hydroxybutyrate (BHB, C), triglycerides (D) and cholesterol (E) of dams (grey) and calves (white) after parturition and before calves had colostrum intake. Dams received glyphosate-contaminated (GLY groups) or control (CON groups) rations in combination with low (LC, 30%) or high (HC, 60%) concentrate feed proportions (CFP) for 16 weeks in mid- and late-lactation and during early gestation (dams: CON_HC_, n = 9; CON_LC_, n = 11; GLY_HC_, n = 10; GLY_LC_, n = 9; calves: CON_HC_, n = 9; CON_LC_, n = 11; GLY_HC_, n = 9; GLY_LC_, n = 9). Values are represented as non-transformed (square) or Box-Cox back-transformed (triangle) Lsmeans with 95% confidence intervals, while violin plots depict distribution of data. * shows significant effects; significant p-values for glucose of dams (p_GLY_ = 0.027, A) and NEFA in calves (p_GLY_ = 0.016, B). For all other variables: p>0.05 (GLY, CFP, GLY*CFP).

After parturition, serum glucose of dams had greater levels in CON groups than in GLY groups (p_GLY_ = 0.027, Fig 1A). NEFA, BHB, triglyceride, and cholesterol levels remained unaffected by dietary treatments of dams during early gestation (Fig 1B–1E). While chloride (p_GLY_ = 0.005), sodium (p_GLY_ = 0.025), and creatinine (p_GLY_ = 0.043) was greater in CON groups than in GLY groups, for urea highest levels in GLY_LC_ and lowest levels in GLY_HC_ were detected (p_GLY*CFP_ = 0.03; p_CFP_ = 0.041, Table 2). Phosphorus levels were highest in GLY_LC_, followed by CON_HC_, GLY_HC_ and CON_LC_ (p_GLY*CFP_ = 0.048), while activities of AST were lowest in GLY_HC_ (p_GLY*CFP_ = 0.031). Levels of albumin, total protein, total bilirubin, uric acid, calcium, potassium and activities of ALT, AP, GGT and GLDH were not influenced by dietary treatment of dams during early gestation shortly after parturition (Table 2).

**Table 2:** Influences of glyphosate residues and different concentrate feed proportions on serum metabolites in dams and calves after parturition and before calves had colostrum intake. Experimental diets were fed for 16 weeks in mid- and late-lactation and during early gestation.

		Group of dams				p-value		
	Animal	CON_HC_	CON_LC_	GLY_HC_	GLY_LC_	GLY	CFP	GLY*CFP
Albumin [g/L]	Dams	32.48 _[31.03, 33.93]_	33.29 _[31.98, 34.60]_	32.11 _[30.74, 33.49]_	32.39 _[30.94, 33.84]_	0.365	0.436	0.700
	Calves	27.21 _[26.19, 28.23]_	28.03 _[27.11, 28.96]_	26.45 _[25.43, 27.47]_	27.13 _[26.11, 28.15]_	0.099	0.134	0.883
Total protein [g/L]	Dams	66.64 _[61.78, 71.48]_	69.44 _[65.05, 73.83]_	63.41 _[58.81, 68.02]_	67.99 _[63.14, 72.84]_	0.318	0.118	0.702
	Calves	42.7 _[40.67, 44.73]_	44.55 _[42.71, 46.38]_	41.45 _[39.42, 43.48]_	42.24 _[40.21, 44.27]_	0.077	0.185	0.590
Total bilirubin [μmol/L]	Dams^#^	6.56 _[4.72, 9.41]_	5.45 _[4.09, 7.43]_	7.08 _[5.14, 10.03]_	6.50 _[4.67, 9.31]_	0.442	0.406	0.750
	Calves^#^	6.27 _[4.30, 9.49]_	6.39 _[4.53, 9.30]_	4.41 _[3.12, 6.43]_	7.43 _[5.02, 11.47]_	0.564	0.153	0.183
AP [μkat/L]	Dams	1.28 _[1.02, 1.53]_	1.23 _[1.00, 1.46]_	1.30 _[1.06, 1.55]_	1.17 _[0.91, 1.43]_	0.905	0.460	0.722
	Calves^#^	3.45 _[2.45, 5.02]_	4.09 _[2.96, 5.83]_	3.2 _[2.28, 4.62]_	2.65 _[1.92, 3.76]_	0.141	0.931	0.297
AST [μkat/L]	Dams^#^	1.31 _[1.17, 1.53]_	1.25 _[1.14, 1.40]_	1.16 _[1.07, 1.28]_	1.42 _[1.25, 1.72]_	0.894	0.184	**0.031**
	Calves^#^	0.32 _[0.26, 0.42]_	0.34 _[0.28, 0.43]_	0.35 _[0.28, 0.47]_	0.33 _[0.27, 0.44]_	0.720	0.960	0.687
ALT [μkat/L]	Dams^#^	0.42 _[0.37, 0.48]_	0.42 _[0.37, 0.47]_	0.42 _[0.37, 0.47]_	0.42 _[0.37, 0.48]_	0.988	0.945	0.818
	Calves	0.17 _[0.14, 0.19]_	0.15 _[0.13, 0.18]_	0.18 _[0.15, 0.21]_	0.17 _[0.14, 0.19]_	0.372	0.374	0.998
GGT [μkat/L]	Dams	0.43 _[0.37, 0.46]_	0.40 _[0.35, 0.46]_	0.43 _[0.37, 0.49]_	0.45 _[0.39, 0.51]_	0.495	0.890	0.427
	Calves	0.26 _[0.20, 0.33]_	0.3 _[0.24, 0.36]_	0.24 _[0.17, 0.30]_	0.2 _[0.17, 0.30]_	0.173	0.620	0.525
GLDH [μkat/L]	Dams	0.17 _[0.09, 0.25]_	0.18 _[0.11, 0.24]_	0.16 _[0.08, 0.23]_	0.25 _[0.04, 0.17]_	0.442	0.197	0.258
	Calves^#^	0.05 _[0.03, 0.07]_	0.05 _[0.04, 0.07]_	0.05 _[0.03, 0.07]_	0.05 _[0.04, 0.07]_	0.746	0.771	0.974
Urea [mmol/L]	Dams	2.09 _[1.48, 2.71]_^ab^	2.05 _[1.49, 2.61]_^ab^	1.57 _[0.98, 2.16]_^a^	2.85 _[2.24, 3.47]_^b^	0.638	**0.041**	**0.030**
	Calves^#^	1.7 _[1.36, 2.22]_	1.63 _[1.34, 2.05]_	1.35 _[1.12, 1.69]_	2.07 _[1.60, 2.82]_	0.906	0.103	**0.049**
Uric acid [μmol/L]	Dams^#^	30.07 _[25.85, 30.7]_	30.20 _[26.32, 35.00]_	27.70 _[24.13, 32.13]_	29.95 _[25.75, 35.25]_	0.542	0.577	0.617
	Calves^#^	53.88 _[42.83, 68.73]_	47.4 _[38.74, 58.63]_	59.59 _[47.10, 76.52]_	46.84 _[37.52, 59.24]_	0.703	0.111	0.625
Calcium [mmol/L]	Dams^#^	1.63 _[1.23, 2.0]_	1.77 _[1.43, 2.11]_	1.77 _[1.40, 2.11]_	2.13 _[1.76, 2.48]_	0.168	0.156	0.535
	Calves^#^	3.09 _[2.91, 3.15]_	3.06 _[2.89, 3.20]_	3.06 _[2.86, 3.22]_	3.02 _[2.82, 3.18]_	0.688	0.693	0.966
Chloride [mmol/L]	Dams	111.24 _[108.85, 113.64]_	109.76 _[107.59, 111.93]_	108.05 _[105.78, 110.33]_	106.20 _[103.80, 108.60]_	**0.005**	0.152	0.871
	Calves	100.67 _[98.82, 102.53]_	102.19 _[100.51, 103.86]_	100.22 _[98.37, 102.08]_	100.0 _[98.14, 101.85]_	0.148	0.475	0.335
Potassium [mmol/L]	Dams^#^	4.39 _[3.87, 5.18]_	4.13 _[3.73, 4.69]_	4.02 _[3.63, 4.57]_	3.89 _[3.52, 4.41]_	0.228	0.437	0.865
	Calves^#^	4.39 _[4.22, 4.60]_^a^	4.8 _[4.57, 5.71]_^ab^	5.17 _[4.81, 5.71]_^b^	4.75 _[4.50, 5.07]_^ab^	**0.011**	0.608	**0.004**
Phosphorus [mmol/L]	Dams	1.28 _[0.99, 1.57]_	1.01 _[0.74, 1.27]_	1.04 _[0.76, 1.31]_	1.33 _[1.04, 1.62]_	0.770	0.948	**0.048**
	Calves^#^	2.84 _[2.36, 3.58]_	2.99 _[2.50, 3.72]_	2.48 _[2.10, 3.02]_	2.44 _[2.08, 2.97]_	0.078	0.861	0.739
Sodium [mmol/L]	Dams	147.17 _[145.05, 149.29]_	145.95 _[144.02, 147.87]_	144.90 _[142.88, 146.91]_	143.51 _[141.38, 145.63]_	**0.025**	0.203	0.935
	Calves	139.59 _[137.51, 141.67]_	140.94 _[139.06, 142.82]_	138.83 _[136.761, 140.91]_	138.52 _[136.44, 140.60]_	0.121	0.610	0.412
Creatinine [μmol/L]	Dams	117.01 _[105.50, 128.53]_	113.44 _[103.02, 123.85]_	109.86 _[98.93, 120.78]_	97.58 _[86.07, 109.10]_	**0.043**	0.156	0.432
	Calves^#^	214.38 _[164.74, 280.0]_	212.29 _[166.74, 270.29]_	227.68 _[174.33, 297.37]_	206.53 _[158.13, 269.74]_	0.899	0.679	0.735

Values are presented as non-transformed or Box-Cox back-transformed (#) Lsmeans with 95% confidence limits [lower limit, upper limit]. Different superscripted letters indicate significant differences between groups. AP = alkaline phosphatase; AST = aspartate aminotransferase; ALT = alanine aminotransferase; CFP = concentrate feed proportions; CON = control; GGT = γ-glutamyltransferase; GLDH = glutamate dehydrogenase; GLY = glyphosate; HC = high concentrate feed proportions; LC = low concentrate feed proportions; Dams: CON_HC_, n = 9; CON_LC_, n = 11; GLY_HC_, n = 10; GLY_LC_, n = 9; Calves: CON_HC_, n = 9; CON_LC_, n = 11; GLY_HC_, n = 9; GLY_LC_, n = 9.

In calves, levels of blood glucose, cholesterol, triglycerides, and BHB remained unaffected by treatments of dams (Fig 1A and 1C–1E), while lower levels of blood NEFA in calves of GLY groups compared to those of CON groups (p_GLY_ = 0.016, Fig 1B) were detected. Additionally, a time-dependent increase of NEFA levels within the first 105 minutes after birth were observed, which is reflected by a significant Spearman´s rank correlation (R = 0.76, p<0.001; S1A Fig). Except for blood potassium and urea levels, no influence of GLY or CFP was observed for any other analyzed clinical-chemical trait in newborn calves (Table 2). Potassium levels were highest in GLY_HC_ followed by CON_LC_, GLY_LC_ and CON_HC_ (p_GLY*CFP_ = 0.004; p_GLY_ = 0.011), while urea levels showed highest levels in GLY_LC_ and lowest levels in GLY_HC_ (p_GLY*CFP_ = 0.049, Table 2, S1B Fig).

### Hematology and antioxidative status

In dairy cows, red cell distribution width was higher in LC groups than in HC groups (p_CFP_<0.001) and plateletcrit levels were highest in CON_HC_ and GLY_LC_ (p_GLY*CFP_ = 0.038), while erythrocytes, platelets, hemoglobin, and other related indices remained unaffected by dietary treatment of dams (S2 Table). After parturition, activities of the antioxidative enzymes GPx and SOD in erythrocytes (S2 Table) as well as white blood profile of dams showed no influences of dietary treatment (CFP, GLY) during gestation (S3 Table).

**Fig 2:**
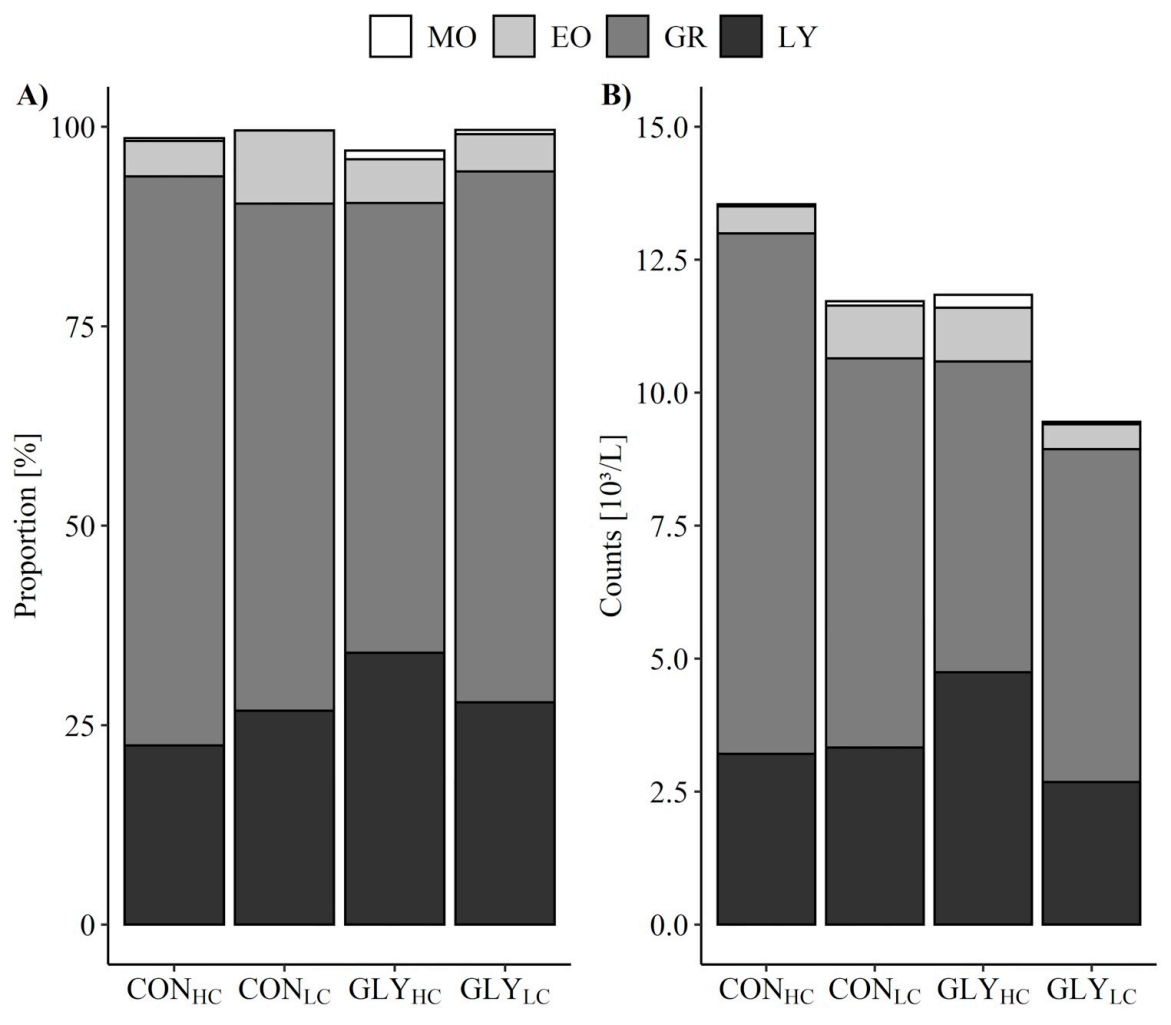
Influence of glyphosate residues and concentrate feed proportions on white blood profile in maternally exposed calves after parturition. Depicted are proportions (A) and cell counts (B) of lymphocytes (LY), granulocytes (GR), eosinophils (EO) and monocytes (MO) (decreasing shades of black to white) and white blood cell count as sum of cell counts (leukocytes) after parturition and before colostrum intake. Dams received glyphosate-contaminated (GLY groups) or control (CON groups) rations in combination with low (LC, 30%) or high (HC, 60%) concentrate feed proportions (CFP) for 16 weeks in mid- and late-lactation and during early gestation (calves: CON_HC_, n = 8; CON_LC_, n = 10; GLY_HC_, n = 9; GLY_LC_, n = 9). Values are presented as Box-Cox back-transformed (proportions of LY, GR, EO, MO) Lsmeans or as means (cell counts of LY, GR, EO, MO and leukocytes). (A): significant p-values for LY (p_GLY_ = 0.020), GR (p_GLY*CFP_ = 0.009); (B): significant p-values for leukocytes (GLY = 0.028) and GR (p_GLY_ = 0.032). For all other variables: p>0.05 (GLY, CFP, GLY*CFP).

In newborn calves, no influences of maternal GLY or CFP treatments during gestation were observed for any of the analyzed parameters regarding red blood profile, platelets, related indices, and erythrocyte antioxidative status (S2 Table), while red cell distribution width was lower in GLY groups (p_GLY_ = 0.047) and platelet distribution width was lower in LC groups (p_CFP_ = 0.029) compared to their respective groups (CON and HC). With regard to the white blood profile of calves after birth, highest proportion of lymphocytes was detected in calves of group GLY_HC_ followed by GLY_LC,_ CON_LC_, and CON_HC_ (p_GLY_ = 0.02, Fig 2A), while leukocyte count (white blood cells) was highest in CON_HC_ and lowest in GLY_LC_ (p_GLY_ = 0.028, Fig 2B). Granulocyte counts were higher in CON groups than in GLY groups (p_GLY_ = 0.032, Fig 2B), while granulocyte proportions showed lowest levels in GLY_HC_, increasing in CON_LC_, GLY_LC_, CON_HC_ (p_GLY*CFP_ = 0.009, Fig 2A). However, cell counts and proportions of monocytes and eosinophils remained unaffected by treatment of dams during gestation for 16 weeks (Fig 2).

### Gene expression analysis in leukocytes

After parturition, expression levels of all genes of interest in blood leukocytes of cows remained unaffected by the treatments (GLY, CFP) during early gestation (S4 Table).

In blood leukocytes of calves, mRNA abundances of toll like receptor 4 (*TLR4*) were higher in HC groups compared to LC groups (p_CFP_ = 0.037), while free fatty acid receptor (*FFAR2*), G protein-coupled receptor 84 (*GPR84*), *GPX1*, neutrophil cytosolic factor 1 (*NCF1*), NFKB inhibitor alpha (*NFKBIA*), nuclear receptor subfamily 3 group C member 1 (*NR3C1*), Rac family small GTPase 2 (*RAC2*), RELA proto-oncogene, NF-kB subunit (*RELA*) and *SOD2* remained unaffected by treatment of dams (Table 3).

**Table 3:** Effects of glyphosate and different concentrate feed proportions in cow’s rations on mRNA abundances of genes of interest in blood leukocytes of calves after birth and before colostrum intake. Experimental diets were fed for 16 weeks in mid- and late-lactation and during early gestation.

	Experimental group of dams				p-value		
	CON_HC_ (n = 9)	CON_LC_ (n = 11)	GLY_HC_ (n = 9)	GLY_LC_ (n = 9)	GLY	CFP	GLY* CFP
*FFAR2*	0.14 _[0.09, 0.22]_	0.12 _[0.08, 0.19]_	0.12 _[0.08, 0.19]_	0.13 _[0.08, 0.20]_	0.870	0.879	0.723
*GPR84*	0.40 _[0.22, 0.72]_	0.23 _[0.14, 0.40]_	0.28 _[0.16, 0.51]_	0.28 _[0.15, 0.50]_	0.745	0.327	0.353
*GPX1*	0.72 _[0.57, 0.92]_	0.59 _[0.48, 0.74]_	0.68 _[0.53, 0.86]_	0.63 _[0.49, 0.80]_	0.965	0.249	0.585
*NCF1*	0.54 _[0.39, 0.75]_	0.37 _[0.28, 0.50]_	0.45 _[0.33, 0.63]_	0.43 _[0.31, 0.59]_	0.913	0.175	0.321
*NFKBIA*	0.85 _[0.65, 1.10]_	0.65 _[0.52, 0.82]_	0.71 _[0.54, 0.91]_	0.65 _[0.51, 0.85]_	0.476	0.187	0.460
*NR3C1*	0.83 _[0.65, 1.06]_	0.65 _[0.52, 0.81]_	0.81 _[0.63, 1.03]_	0.71 _[0.56, 0.91]_	0.787	0.122	0.609
*RAC2*	0.71 _[0.56, 0.89]_	0.61 _[0.50, 0.75]_	0.64 _[0.51, 0.80]_	0.56 _[0.44, 0.70]_	0.394	0.199	0.970
*RELA*	0.80 _[0.60, 1.06]_	0.65 _[0.50, 0.84]_	0.70 _[0.53, 0.93]_	0.65 _[0.49, 0.87]_	0.645	0.335	0.632
*SOD2*	0.33 _[0.23, 0.47]_	0.21 _[0.15, 0.28]_	0.22 _[0.16, 0.32]_	0.22 _[0.15, 0.31]_	0.313	0.149	0.199
*TLR4*	0.69 _[0.49, 0.97]_	0.42 _[0.31, 0.57]_	0.54 _[0.39, 0.76]_	0.44 _[0.32, 0.62]_	0.578	**0.037**	0.360

Values are presented as log back-transformed Lsmeans with 95% confidence limits [lower limit, upper limit]. CFP = concentrate feed proportion; CON = control; GLY = glyphosate; *FFAR2* = free fatty acid receptor 2; *GPR84* = G protein-coupled receptor 84; *GPX1* = glutathione peroxidase 1; HC = high CFP; LC = low CFP; *NCF1* = neutrophil cytosolic factor 1; *NFKBIA* = NFKB inhibitor alpha; NR3C1 = nuclear receptor subfamily 3 group C member 1; *RAC2* = Rac family small GTPase 2; *RELA* = RELA proto-oncogene, NF-kB subunit; *SOD2* = superoxide dismutase 2; *TLR4* = toll like receptor 4.

### Functional properties of immune cells

**Fig 3:**
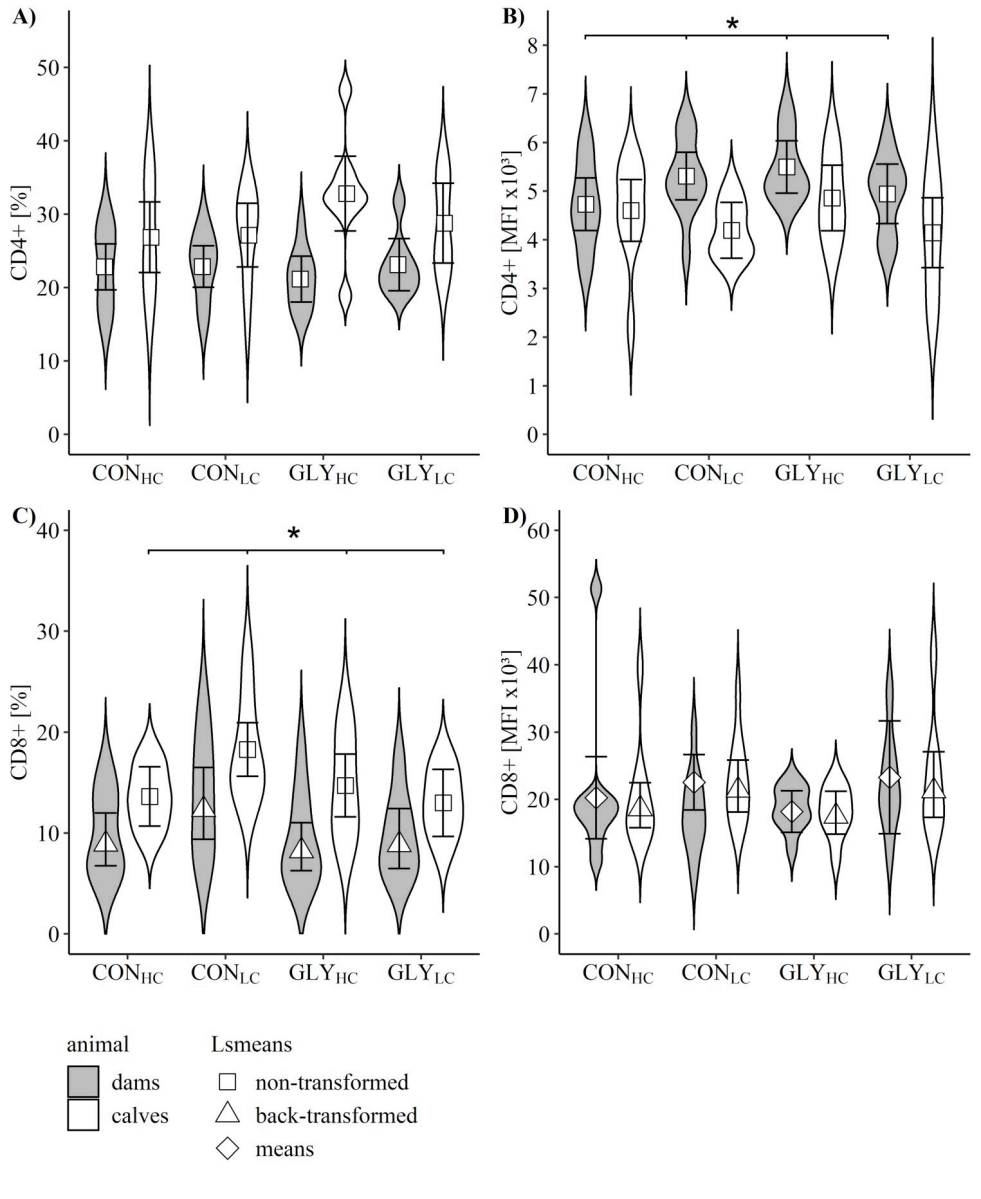
Relative proportions and mean fluorescence intensities (MFI) of CD4+ and CD8+ cells of total peripheral blood mononuclear cells in dams and calves after parturition. Experimental diets were fed for 16 weeks diets fed for 16 weeks in mid- and late-lactation and during early gestation. Depicted are proportions of CD4+ (A) and CD8+ (C) cells as well as respective MFI (CD4+: B; CD8+: D) of dams (grey) and calves (white) after parturition and before calves had colostrum intake. Dams received glyphosate-contaminated (GLY groups) or control (CON groups) rations in combination with low (LC, 30%) or high (HC, 60%) concentrate feed proportions (CFP) for 16 weeks in mid- and late-lactation and during early gestation (dams: CON_HC_, n = 9; CON_LC_, n = 11; GLY_HC_, n = 10; GLY_LC_, n = 9; calves: CON_HC_, n = 9; CON_LC_, n = 11; GLY_HC_, n = 8; GLY_LC_, n = 7). Values are represented as non-transformed (square), Box-Cox back-transformed (triangle) Lsmeans or means (diamond) with 95% confidence interval, while violin plots depict distribution of data. * shows significant effects; significant p-values for CD4+ (MFI x 10^3^) of dams (p_GLY*CFP_ = 0.042, B) and CD8+ proportions of calves (p_GLY*CFP_ = 0.039, C). For all other variables: p>0.05 (GLY, CFP, GLY*CFP).

Proportions of subpopulations of lymphocytes expressing CD4 molecule and CD8 expressing cells of dams were not influenced by dietary treatment (Fig 3A and 3C), while MFI of CD4+ cells were highest in GLY_HC_ and lowest in CON_HC_ (p_GLY*CFP_ = 0.042, Fig 3B).

In newborn calves, proportions of CD8+ cells were elevated in CON_LC_ followed by GLY_HC_, CON_HC_ and GLY_LC_ (p_GLY*CFP_ = 0.039, Fig 3C), while proportions of CD4+ and respective MFI of both cell types remained unaffected by dietary treatment of the dams (Fig 3A, 3B and 3D).

Measurements of intracellular ROS production in PMN revealed no influences of GLY or CFP in dams and calves after parturition and before calves had colostrum intake on proportions and MFI of basal and stimulated ROS forming cells (S5 Table).

**Fig 4:**
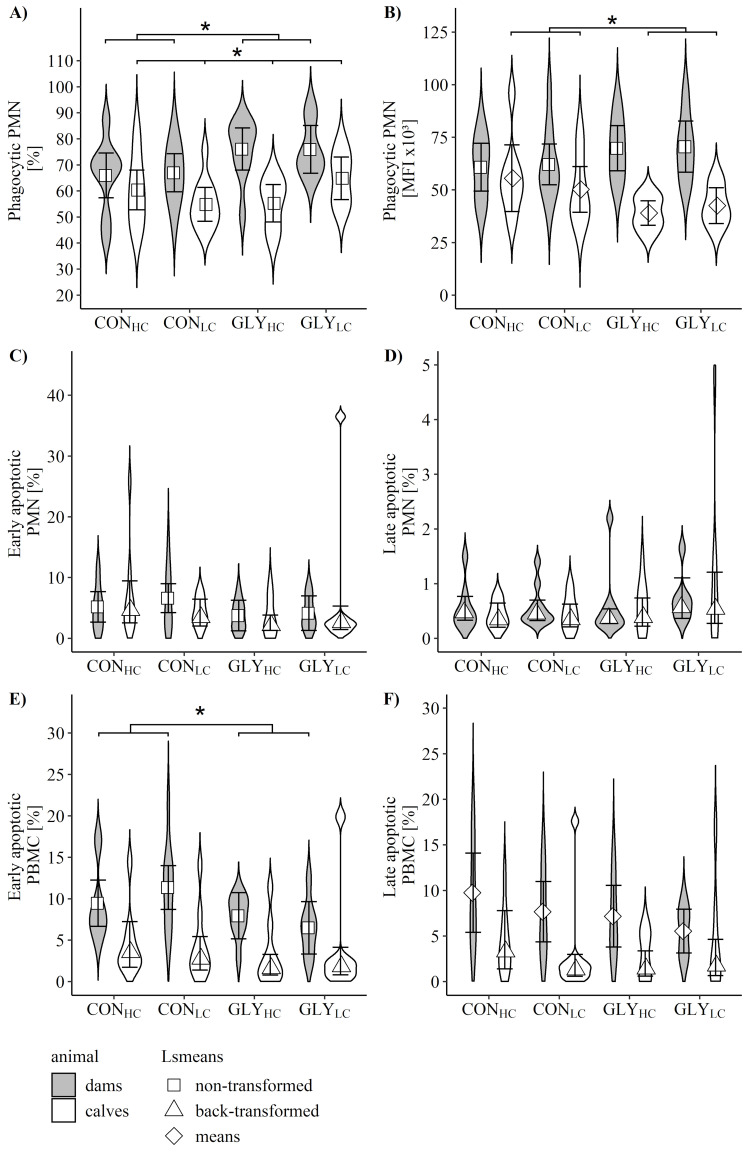
Phagocytic activity, early and late apoptotic polymorphonuclear leukocytes and peripheral blood mononuclear cells in dams and calves after parturition. Depicted are proportions (A) and mean fluorescence intensity (MFI; B) of phagocytic polymorphonuclear leukocytes (PMN), proportions of early and late apoptotic PMN (C, D) and peripheral blood mononuclear cells (PBMC; E, F) of dams (grey) and calves (white) after parturition and before calves had colostrum intake. Dams received glyphosate-contaminated (GLY groups) or control (CON groups) rations in combination with low (LC, 30%) or high (HC, 60%) concentrate feed proportions (CFP) for 16 weeks in mid- and late-lactation and during early gestation (Dams: CON_HC_, n = 9; CON_LC_, n = 11; GLY_HC_, n = 10; GLY_LC_, n = 9; Calves: CON_HC_, n = 8 (9 samples for apoptotic cells measurements); CON_LC_, n = 11 (10 samples for apoptotic cells measurements); GLY_HC_, n = 9; GLY_LC_, n = 7). Values are represented as non-transformed (square), Box-Cox back-transformed (triangle) Lsmeans or means (diamond) with 95% confidence interval, while violin plots depict distribution of data. * shows significant effects; significant p-values for proportions of phagocytic PMN of dams (p_GLY_ = 0.026, A) and calves (p_GLY*CFP_ = 0.045, A), for capacity of phagocytic PMN of calves (p_GLY_ = 0.030, B) as well as proportions of early apoptotic PBMC of dams (p_GLY_ = 0.030, E). For all other variables: p>0.05 (GLY, CFP, GLY*CFP).

Measurements of proportions and capacity of phagocytic cells revealed higher proportions in GLY groups than in CON groups of dams (p_GLY_ = 0.026, Fig 4A). In calves, proportion of phagocytic cells was highest in GLY_LC_ followed by CON_HC_, GLY_HC_ and CON_LC_ (p_GLY*CFP_ = 0.045, Fig 4A), while capacity was increased in the CON groups (p_GLY_ = 0.030, Fig 4B).

Proportions of early and late apoptotic (Fig 4C–4F) as well as necrotic PMN and PBMC (S6 Table) in calves displayed no significant effects related to the treatments of dams. In dams, with exception of an elevated proportion of early apoptotic PBMC in the CON groups (p_GLY_ = 0.03), neither the proportions of apoptotic (early and late, Fig 4C–4F) nor necrotic blood cells (PMN and PBMC, S6 Table) were affected by dietary treatments of dams during early gestation.

### DNA damage indicators

Results of the comet assay of blood leukocytes showed no influence of dietary treatments of dams on Tail DNA and Olive tail moment of dams and calves (S7 Table).

## Discussion

During gestation, the development of the fetus as well as the postnatal health of the offspring might be influenced by maternal nutrition [1]. Although nutrient requirements of the developing fetus vary in different stages of embryonic life, maternal nutrition influences pre- and postnatal development depending on the fetal stage [1]. From a quantitative viewpoint, nutrient requirements are low during the first months of gestation and increase with exponential growth of the fetus during the last 2 months of parturition [1]. In parallel, maternal exposure to various stressors, such as environmental conditions or exposure to toxic substances, can affect fetal development adversely [1, 2]. Therefore, next to putative effects of GLY as active substance in non-selective herbicides on the orally exposed dam [7], effects of *in utero* GLY exposure on livestock’s offspring, such as pigs [14, 15] or dairy cows, are of scientific interest. As effects of GLY were reported for offspring of pigs [14] and mice [17], developing calves might also be affected by GLY residues in the rations of dairy cows during gestation. For this reason, pregnant cows used in the present study originated from a feeding trial recently described by several authors [6, 18–20]. The number of cows used in this study does not represent a sample size that enabled the detection of significant treatment effects for a number of parameters. Therefore, the interpretability is limited for those parameters not reaching significance. However, we consider that this data and the supporting metabolic measures, will provide others with data that may be pooled in future studies on exposure of cows to GLY. Due to agricultural conditions, the number of pregnant animals and consequently the number of calves was limited. Dams of this study were exposed to GLY residues for 16 weeks at different stages of early gestation followed by a depletion period. This period was included due to technical reasons, such as feed adaptation during dry period, limiting the putative experimental period within a dairy cow herd with seasonal calving or limited production of experimental feedstuff. Despite using the same feedstuff batches over the experimental period, application mode might have influenced as a confounding variable, since treated crops were located at different sections of the FLI Research Station.

For the subset of animals used in the present study, average daily GLY exposure was 1.2 (CON_LC_), 1.1 (CON_HC_), 112.6 (GLY_LC_) and 130.3 μg/kg body weight (GLY_HC_). Higher GLY exposure in GLY_HC_ might be a consequence from increased dry matter intake during the first weeks of feeding compared to GLY_LC_. However, the present study investigated the occurrence of putative long-term GLY effects on dam and offspring, which might lead to pathological changes that persist until parturition irrespective of a depletion period after GLY exposure.

In general, transitional cows are faced with nutritional, physiological, and energetical challenges resulting from/in physiological adaptions and dynamic changes associated with the homeostatic and homeorhetic adaptations around calving as reviewed [42, 43]. Next to e.g., impairments of the immune system and increased oxidative stress, negative energy balance characterize the transition period [42, 43]. Until parturition, energy is required for maintenance purposes, colostrogenesis and fetus growth, whereas *postpartum* high energy needs for the onset of lactogenesis on the one hand, and only slowly increasing feed and energy intake on the other hand lead to energy deficits [44–46]. This negative energy balance *postpartum* is in general paralleled by an increase of blood NEFA and BHB levels [44]. However, parturition itself is a stressful and energy consuming event [47]. In the present trial, no influence of GLY or CFP fed during early gestation was observed on dams’ blood levels of BHB, NEFA, cholesterol, or triglycerides, indicative for energy metabolism, while glucose levels were slightly decreased in GLY groups compared to CON groups after parturition. Although *postpartum* significant effects of GLY feeding in early gestation were observed for blood glucose, chloride, sodium, creatinine concentrations, and in combination with different CFP for phosphorus concentrations and AST activity in dams, it must be noted that a recent study has demonstrated characteristic time-dependent physiological adaptions after parturition [48, 49]. These results urge attention to the exact sampling time in relation to the calving process. For experimental reasons, in this study, sampling after calving differed from 5 to 345 min and, due to this time factor, an additional cow-specific variation existed, which is difficult to interpret. Furthermore, levels were comparable to values measured in post-calving cows at the same research institute (glucose, phosphorus, chloride, sodium [49]) or comparable to reference values (creatinine, AST [50]) and thus corresponding to physiological values.

However, a focus of the present study was the investigation of putative effects of an *in utero* GLY exposure on the offspring of GLY-exposed dams. In general, no evidence for malformations in newborn calves could be collected, which is in accordance to observations in Wistar rats [9] and contrary to those in pigs [14]. While the rats were treated with 70 mg GLY/kg BW/d during gestational day 5 until postnatal days 15 or 60 [9], malformations in piglets were associated to 0.87 to 1.13 mg GLY/kg feed fed to sows. However, no adequate data regarding feed analyses or control animals were shown [14]. Absence of GLY effects on offspring’s birth weight in the present study, by contrast, were in accordance to studies in rats treated with 50–1000 mg GLY/kg BW/d during different periods of gestation [9, 10, 16].

Immediately after birth, neonatal calves need to adapt to various challenges such as changing from continuous maternal nutrient supply to discontinuous energy intake via colostrum or milk [51]. As reviewed [51], in neonatal calves hepatic glycogen is usually the first energy source leading to euglycemia after birth, while plasma glucose levels persist on levels higher than 3.5 mmol/L if no milk intake occurs within 24h after birth. In the present study, NEFA levels were lower in calves of GLY groups compared to those of CON groups which possibly resulted from coincidentally shorter timeframes between birth and sample collection in GLY groups than in CON groups. This is reflected in time-dependent NEFA levels within the first 105 minutes after birth and before colostrum intake (Spearman´s rank correlation R = 0.76, p<0.001, S1A Fig). This time-dependent pattern indicated the ability to provide NEFA for energy supply by fat mobilization in agreement with previous observations [51–53].

This increase in blood NEFA levels, as indicators for fat mobilization and/or elevated stress levels after birth due to delayed colostrum intake, might affect immune responses of calves [44, 54]. Thus, and in order to analyze putative GLY associated effects, hematological parameters such as leukocytes counts and their subtypes were evaluated.

In contrast to red blood profile and platelet-associated parameters, some parameters within the white blood profile showed significant GLY effects in calves. However, regarding total leukocytes, slightly lower mean counts observed in GLY groups (10.6 ± 5.2 10^3^/μL) compared to CON groups (12.6 ± 3.7 10^3^/μL) were comparable to values in newborn calves irrespective of treatment (10.03 ± 1.18 10^9^/L; [55]). Additionally, GLY effects on proportions of lymphocytes and granulocytes were significant but weak and showed only slight differences between mean proportions when contrasting GLY groups and CON groups (lymphocytes: 30.7% ± 1.6 vs. 24.5% ± 1.1; granulocytes: 61.8% ± 6.6 vs. 67.6% ± 5.7). Comparing the results found in newborn calves with those from adult cattle, different CFP and/or experimental time influenced total lymphocyte and granulocyte counts in dairy cows [20]. No adverse effects of GLY were observed upon these practical GLY exposure conditions (average GLY exposure: 122.7 μg GLY/kg BW/d [20]).

Selected gene expression analyses were performed to substantiate findings of the present experiment (NEFA receptor related genes) or findings putatively associated to GLY exposure. Also genes related to oxidative status were analyzed for expression on mRNA level since differences in mRNA levels had been observed in GLY-treated rats as discussed below [56]. In the present study, analyzed parameters related to blood’s oxidative status (SOD and GPx activities, FRAP) of neonatal calves were not influenced by dietary treatment. Additionally, leukocyte expression of oxidative status related genes *SOD2*, *GPX1*, *NCF1*, *RAC2* was not influenced by dietary treatment of the dams. While these results were in accordance with SOD and GPx activities mentioned above, other authors reported significant changes in expression levels of oxidative stress related genes in the brain of male Wistar rats at postnatal day 90 after maternal exposure to Glyphosate Roundup® Transorb from gestational day 19 until birth [56]. While increasing expression levels of *GPX1* in the cerebellum of animals treated with 50 mg GLY/kg/d were observed, *SOD1* and *SOD2* expression levels remained unaffected in the cortex and cerebellum [56]. For *SOD2* this is in accordance with results in leukocytes of newborn calves. De Souza et al. [56] used GLY concentrations that were elevated by orders of magnitude in comparison to the present study. Additionally, laboratory animals and brain samples were used instead of leukocytes and rats were exposed until the end of gestation without depletion period, possibly explaining differences in observations.

As reviewed [44, 54], different blood metabolites have an influence on immune cells in different ways. Glucose serves as the preferred metabolic fuel, while other metabolites have immunostimulatory or immunosuppressive effects (NEFA [57, 58]). Irrespective of the time-related pattern, a putative effect of GLY on NEFA levels might have consequently affected functional properties of blood cells such as ROS production ability of bovine PMN [58].

In the present experiment, significant effects of GLY or CFP on proportions and MFI of basal and stimulated ROS producing PMN at parturition were neither observed in newborn calves nor in dams, while a significant interaction between CFP and time was found for stimulated ROS production in dairy cows fed different CFP for 16 weeks [20]. During the depletion period appropriate diets in accordance with the individual milk yield were fed, which might have masked this effect at parturition, while an influence of GLY was not observed at any analyzed time.

In accordance with results in dairy cows upon GLY exposure [20], no genotoxic effects of GLY or CFP were observed in the present study displayed by the absence of DNA damages in bovine leukocytes measured by comet assay or by no adverse influences on apoptotic cells in dams or newborn calves.

To our knowledge, the present experiment is the first study analyzing effects of maternal exposure of GLY in combination with different CFP during early gestation on newborn dairy calves in general and on multiple blood parameters such as functionality of blood cells in particular. It should be mentioned that exposure to conditions during late gestation, where fetal growth is greatest [2], might have led to different results as have been observed and discussed in the present study.

Discrepancies between results of the present and other studies might have occurred due to different subjects in the studies (cattle vs. laboratory animals and cell types), analyzed tissues/matrices (blood vs. e.g. brain), experimental designs (*ex vivo* and *in vivo* vs. *in vitro*), used dosages or different surfactants/GBHs in the glyphosate formulation.

However, there were additional limitations regarding the present trial which should be kept in mind when interpreting the results. In contrast to other studies, which analyzed effects of GLY or GBHs in the offspring during or shortly after maternal exposure (postnatal) without depletion time [9–11, 17, 56], dams of this study were exposed to GLY residues for 16 weeks at different stages of early gestation followed by a depletion period. For this reason, the beginning of maternal and, therefore, fetal exposure of 16 weeks varied between 21 and 107 days representing different stages of fetal development. Thus, we cannot exclude that a GLY exposure in earlier states of embryonic and fetal development could have exerted adverse effects. Although some traits showed GLY effects, possible effects of partus itself could not be excluded since parturition is a stressful event for the dam and her calf. In the present study, different calving ease did not show a balanced distribution within the groups. While two animals in GLY_HC_ and CON_LC_ needed much assistance, including technical equipment, for all other animals no or little assistance was provided. Therefore, measurements of stress-related parameters such as blood cortisol levels, more detailed evaluation of calving ease as well as constant sampling times after parturition should be addressed in further studies to better distinguish between effects of GLY or effects of the parturition or a combination of both.

In summary, the magnitude of GLY effects observed for analyzed traits was low and might not be reproducible. Since most effects were not significantly different between groups in a post-hoc test, the magnitude of GLY effects was hardly pronounced and ranged within levels already observed independent of xenobiotic exposure in other studies questioning the toxicological relevance of these effects. Additionally, given the large number of tests conducted, there is considerable potential for type I statistical error. Finally, a possibly existing relevance of GLY effects on newborn calves and their dams should be addressed in further studies including exposure studies during complete or at least late gestation in combination with greater numbers of animals and synchronized time periods between birth and sample collection.

## Conclusions

The present study aimed to investigate putative long-term effects of GLY residues in dairy cow rations for 16 weeks during early gestation on the health of dairy cows and their offspring after parturition. Any effects of GLY, CFP or interactions were weak and levels of parameters were comparable to already observed levels from other studies. Additionally, these effects were possibly a function of Type I statistical error given the large number of tests conducted. Therefore, it can be concluded that under applied experimental conditions, no evidence of teratogenic or other clear effects of GLY or CFP on analyzed parameters of dams and their newborn calves could be collected at parturition and before calves had colostrum intake or, even if existent, effects are rather not consistent between early gestation and partus.

## Supporting information

S1 Fig Spearman’s rank correlation of serum non-esterified fatty acid (NEFA) levels in neonatal calves after birth and time between birth and blood sampling before colostrum intake (A), as well as of urea levels of calves and dams after parturition (B). Calves were maternally exposed to glyphosate-contaminated (GLY) or control rations (CON) and to high (HC) or low (LC) concentrate feed proportions for 16 weeks in mid- and late-lactation and during early gestation. Spearman´s rank correlation coefficients were calculated for all data (A, B) and additionally excluding data from calves with blood collection >250 min after birth (A).
(TIF)

S1 Table Characterization of primers used in quantitative real-time PCR. (XLSX)

S2 Table Red blood profile, platelets and related indices and oxidative status in dams and calves after parturition and before colostrum intake after feeding dams 16 weeks rations with glyphosate residues and different concentrate feed proportions in mid- and late-lactation and during early gestation. (XLSX)

S3 Table Influence of glyphosate residues and different concentrate feed proportions in diets fed for 16 weeks in mid- and late-lactation and during early gestation on white blood profile in dams after parturition. (XLSX)

S4 Table Influence of glyphosate residues and different concentrate feed proportions in diets fed for 16 weeks in mid- and late-lactation and during early gestation on leukocyte gene expression in dams after parturition. (XLSX)

S5 Table Intracellular reactive oxygen species production of polymorphonuclear leukocytes of dams and calves after parturition and before colostrum intake after feeding dams 16 weeks rations with glyphosate residues and different concentrate feed proportions in mid- and late-lactation and during early gestation. (XLSX)

S6 Table Necrotic polymorphonuclear leukocytes and peripheral blood mononuclear blood cells of dams and calves after parturition and before colostrum intake after feeding dams 16 weeks rations with glyphosate residues and different concentrate feed proportions in mid- and late-lactation and during early gestation. (XLSX)

S7 Table DNA damage indicators in leukocytes of dams and calves after parturition and before colostrum intake after feeding dams 16 weeks rations with glyphosate residues and different concentrate feed proportions in mid- and late-lactation and during early gestation. (XLSX)

## Abbreviations

AP: alkaline phosphatase
ALT: alanine aminotransferase
AST: aspartate aminotransferase
BHB: β-hydroxybutyrate
BW: body weight
CFP: concentrate feed proportions
CON: control
CV: coefficients of variation
DHR: dihydrorhodamine 123
DNA: deoxyribonucleic acid
EDTA: ethylenediaminetetraacetic
EO: eosinophils
*FFAR2*: free fatty acid receptor 2
FITC: fluorescein isothiocyanate
FLI: Friedrich-Loeffler-Institut
FRAP: ferric reducing ability of plasma
GBH: glyphosate-based herbicides
GLDH: glutamate dehydrogenase
GLY: glyphosate
GGT: γ-glutamyltransferase
*GPR84*: G protein-coupled receptor 84
GPx: glutathione peroxidase
*GPX1*: glutathione peroxidase 1
GR: granulocytes
HC: high concentrate feed proportions
LC: low concentrate feed proportions
LY: lymphocytes
MFI: mean fluorescence intensities
MO: monocytes
*NCF1*: neutrophil cytosolic factor 1
NEFA: non-esterified fatty acids
*NFKBIA*: NFKB inhibitor alpha
*NR3C1*: nuclear receptor subfamily 3 group C member 1
PBMC: peripheral blood mononuclear cells
PCR: polymerase chain reaction
PI: propidium iodide
PMN: polymorphonuclear leukocytes
*RAC2*: Rac family small GTPase 2
RELA: RELA proto-oncogene
NF-: kB subunit
RNA: ribonucleic acid
ROS: reactive oxygen species
R123+: rhodamine 123
SOD: superoxide dismutase
*SOD2*: superoxide dismutase 2
*TLR4*: toll like receptor 4
TPA: 12-O-tetradecanoylphorbol-13-acetate
*UCHL*: ubiquitin c-terminal hydrolase L5
*UXT*: ubiquitously expressed prefoldin like chaperone

## References

[1] Funston RN, Larson DM, Vonnahme KA. Effects of maternal nutrition on conceptus growth and offspring performance: Implications for beef cattle production1. J Anim Sci. 2010;88(suppl_13):E205–E15. 19820049 10.2527/jas.2009-2351

[2] Abuelo A. Symposium review: Late-gestation maternal factors affecting the health and development of dairy calves. J. Dairy Sci. 2020;103(4):3882–93. doi: 10.3168/jds.2019-17278 32037167

[3] Duke SO, Powles SB. Glyphosate: a once-in-a-century herbicide. Pest Manag Sci. 2008;64(4):319–25. doi: 10.1002/ps.1518 18273882

[4] Bundesministeriums für Ernährung und Landwirtschaft (2021). Fünfte Verordnung zur Änderung der Pflanzenschutz-Anwendungsverordnung $3 Absatz (5). Bundesgesetzblatt Teil 1 Nr.62 vom 07.09.2021.

[5] Zoller O, Rhyn P, Rupp H, Zarn JA, Geiser C. Glyphosate residues in Swiss market foods: monitoring and risk evaluation. Food Addit Contam Part B Surveill. 2018;11(2):83–91. doi: 10.1080/19393210.2017.1419509 29284371

[6] Schnabel K, Schmitz R, von Soosten D, Frahm J, Kersten S, Meyer U, et al. Effects of glyphosate residues and different concentrate feed proportions on performance, energy metabolism and health characteristics in lactating dairy cows. Arch Anim Nutr. 2017;71(6):413–27. doi: 10.1080/1745039X.2017.1391487 29110579

[7] von Soosten D, Meyer U, Hüther L, Dänicke S, Lahrssen-Wiederholt M, Schafft H, et al. Excretion pathways and ruminal disappearance of glyphosate and its degradation product aminomethylphosphonic acid in dairy cows. J Dairy Sci. 2016;99(7):5318–24. doi: 10.3168/jds.2015-10585 27108173

[8] Steinborn A, Alder L, Michalski B, Zomer P, Bendig P, Martinez SA, et al. Determination of Glyphosate Levels in Breast Milk Samples from Germany by LC-MS/MS and GC-MS/MS. J. Agr. Food Chem. 2016;64(6):1414–21. doi: 10.1021/acs.jafc.5b05852 26808680

[9] Cattani D, Cesconetto PA, Tavares MK, Parisotto EB, De Oliveira PA, Rieg CEH, et al. Developmental exposure to glyphosate-based herbicide and depressive-like behavior in adult offspring: Implication of glutamate excitotoxicity and oxidative stress. Toxicology. 2017;387:67–80. doi: 10.1016/j.tox.2017.06.001 28627408

[10] Dallegrave E, Mantese FD, Oliveira RT, Andrade AJM, Dalsenter PR, Langeloh A. Pre- and postnatal toxicity of the commercial glyphosate formulation in Wistar rats. Archives of Toxicology. 2007;81(9):665–73. doi: 10.1007/s00204-006-0170-5 17634926

[11] Ruuskanen S, Rainio MJ, Uusitalo M, Saikkonen K, Helander M. Effects of parental exposure to glyphosate-based herbicides on embryonic development and oxidative status: a long-term experiment in a bird model. Sci. Rep. 2020;10(1):6349–. doi: 10.1038/s41598-020-63365-1 32286465 PMC7156732

[12] Williams AL, Watson RE, DeSesso JM. Developmental and reproductive outcomes in humans and animals after glyphosate exposure: a critical analysis. J Toxicol Environ Health B Crit Rev. 2012;15(1):39–96. doi: 10.1080/10937404.2012.632361 22202229

[13] Arbuckle TE, Lin Z, Mery LS. An exploratory analysis of the effect of pesticide exposure on the risk of spontaneous abortion in an Ontario farm population. Environ. Health Perspect. 2001;109(8):851–7. doi: 10.1289/ehp.01109851 11564623 PMC1240415

[14] Krüger M, Schrödl W, Pedersen I. Detection of Glyphosate in Malformed Piglets. J Environ Anal Toxicol. 2014;04(05).

[15] Winters JFM, Foldager L, Krogh U, Nørskov NP, Sørensen MT. Impact of glyphosate residues in sow diets on neonatal piglets: tail kinks, stillborn and diarrhoea. Livest. Sci. 2023;269:105172.

[16] Dallegrave E, Mantese FD, Coelho RS, Pereira JD, Dalsenter PR, Langeloh A. The teratogenic potential of the herbicide glyphosate-Roundup® in Wistar rats. Toxicol. 2003;142(1–2):45–52. doi: 10.1016/s0378-4274(02)00483-6 12765238

[17] Ren X, Dai P, Perveen A, Tang Q, Zhao L, Jia X, et al. Effects of chronic glyphosate exposure to pregnant mice on hepatic lipid metabolism in offspring. Environ Pollut. 2019;254(Pt A):112906. doi: 10.1016/j.envpol.2019.07.074 31374489

[18] Billenkamp F, Schnabel K, Hüther L, Frahm J, von Soosten D, Meyer U, et al. No hints at glyphosate-induced ruminal dysbiosis in cows. NPJ Biofilms Microbiomes. 2021;7(1):30. doi: 10.1038/s41522-021-00198-4 33767196 PMC7994389

[19] Heymann A-K, Schnabel K, Billenkamp F, Bühler S, Frahm J, Kersten S, et al. Effects of glyphosate residues and different concentrate feed proportions in dairy cow rations on hepatic gene expression, liver histology and biochemical blood parameters. PLoS One. 2021;16(2):e0246679. doi: 10.1371/journal.pone.0246679 33577576 PMC7880452

[20] Schnabel K, Schmitz R, Frahm J, Meyer U, Breves G, Dänicke S. Functionality and DNA-damage properties of blood cells in lactating cows exposed to glyphosate contaminated feed at different feed energy levels. Arch Anim Nutr. 2020:1–20. 32020815 10.1080/1745039X.2020.1718474

[21] Ackermann W, Coenen M, Schrodl W, Shehata AA, Kruger M. The influence of glyphosate on the microbiota and production of botulinum neurotoxin during ruminal fermentation. Curr Microbiol. 2015;70(3):374–82. doi: 10.1007/s00284-014-0732-3 25407376

[22] Gesellschaft für Ernährungsphysiologie (GfE). Ausschuss für Bedarfsnormen der Gesellschaft für Ernährungsphysiologie: empfehlung zur Energie- und Nährstoffversorgung der Milchkühe und Aufzuchtrinder. Frankfurt: DLG-Verlag (Main). 2001.

[23] Bühler S, Frahm J, Tienken R, Kersten S, Meyer U, Huber K, et al. Effects of energy supply and nicotinic acid supplementation on serum anti-oxidative capacity and on expression of oxidative stress-related genes in blood leucocytes of periparturient primi- and pluriparous dairy cows. J Anim Physiol Anim Nutr (Berl). 2018;102(1):e87–e98. doi: 10.1111/jpn.12705 28439984

[24] Benzie IF, Strain JJ. The ferric reducing ability of plasma (FRAP) as a measure of "antioxidant power": the FRAP assay. Anal. Biochem. 1996;239(1):70–6. doi: 10.1006/abio.1996.0292 8660627

[25] Ye J, Coulouris G, Zaretskaya I, Cutcutache I, Rozen S, Madden TL. Primer-BLAST: A tool to design target-specific primers for polymerase chain reaction. BMC Bioinformatics. 2012;13(1):134. doi: 10.1186/1471-2105-13-134 22708584 PMC3412702

[26] Koressaar T, Remm M. Enhancements and modifications of primer design program Primer3. Bioinformatics. 2007;23(10):1289–91. doi: 10.1093/bioinformatics/btm091 17379693

[27] Untergasser A, Cutcutache I, Koressaar T, Ye J, Faircloth BC, Remm M, et al. Primer3—new capabilities and interfaces. Nucleic Acids Res. 2012;40(15):e115–e. doi: 10.1093/nar/gks596 22730293 PMC3424584

[28] Bühler S, Frahm J, Tienken R, Kersten S, Meyer U, Huber K, et al. Influence of energy level and nicotinic acid supplementation on apoptosis of blood leukocytes of periparturient dairy cows. Vet Immunol Immunopathol. 2016;179:36–45. doi: 10.1016/j.vetimm.2016.07.007 27590424

[29] Singh NP, McCoy MT, Tice RR, Schneider EL. A simple technique for quantitation of low levels of DNA damage in individual cells. Exp Cell Res. 1988;175(1):184–91. doi: 10.1016/0014-4827(88)90265-0 3345800

[30] Końca K, Lankoff A, Banasik A, Lisowska H, Kuszewski T, Góźdź S, et al. A cross-platform public domain PC image-analysis program for the comet assay. Mut. Res. 2003;534(1–2):15–20. doi: 10.1016/s1383-5718(02)00251-6 12504751

[31] Olive PL, Banáth JP, Durand RE. Heterogeneity in Radiation-Induced DNA Damage and Repair in Tumor and Normal Cells Measured Using the "Comet" Assay. Radiat. Res. 1990;122(1):86–94. 2320728

[32] Olive PL, Durand RE. Heterogeneity in DNA damage using the comet assay. Cytom.,: j. Int. Soc. Anal. Cytol. 2005;66(1):1–8. doi: 10.1002/cyto.a.20154 15924303

[33] RStudio Team. RStudio: Integrated Development for R. 1.2.5042 ed. Boston, MA: RStudio, Inc.,; 2020.

[34] R Core Team. R: A language and environment for statistical computing. Vienna, Austria: R Foundation for Statistical Computing; 2020.

[35] Wilke CO. cowplot: Streamlined Plot Theme and Plot Annotations for ’ggplot2’. 2020.

[36] Wickham H. ggplot2: Elegant Graphics for Data Analysis: Springer-Verlag New York; 2016.

[37] Kassambara A. ggpubr: ’ggplot2’ Based Publication Ready Plots2020.

[38] Auguie B. gridExtra: Miscellaneous Functions for "Grid" Graphics2017.

[39] Wickham H, Pedersen TL. gtable: Arrange ’Grobs’ in Tables2019.

[40] Schauberger P, Walker A. openxlsx: Read, Write and Edit xlsx Files. 2021.

[41] Wickham H, Bryan J. Readxl: Read Excel Files. 2019.

[42] Trevisi E, Minuti A. Assessment of the innate immune response in the periparturient cow. Res. Vet. Sci. 2018;116:47–54. doi: 10.1016/j.rvsc.2017.12.001 29223307

[43] Ingvartsen K. Feeding- and management-related diseases in the transition cow: Physiological adaptations around calving and strategies to reduce feeding-related diseases. Anim. Feed Sci. Technol. 2006;126:175–213.

[44] Dänicke S, Meyer U, Kersten S, Frahm J. Animal models to study the impact of nutrition on the immune system of the transition cow. Res. Vet. Sci. 2018;116:15–27. doi: 10.1016/j.rvsc.2018.01.023 29428254

[45] Godden S. Colostrum management for dairy calves. Vet Clin North Am Food Anim Pract. 2008;24(1):19–39. 18299030 10.1016/j.cvfa.2007.10.005 PMC7127126

[46] Barrington GM, McFadden TB, Huyler MT, Besser TE. Regulation of colostrogenesis in cattle. Livest. Prod. Sci. 2001;70(1):95–104.

[47] Hydbring Sandberg E, Madej A, MacDonald E, Drugge-Boholm G, Olsson K. Hormonal changes during parturition in heifers and goats related to the phases and severity of labour. J. Endocrinol. 1999;160:75–85. 9854179 10.1677/joe.0.1600075

[48] Kononov S, Meyer J, Frahm J, Kersten S, Kluess J, Buehler S, et al. Dietary L-Carnitine Affects Leukocyte Count and Function in Dairy Cows Around Parturition. Front. Immunol. 2022;13. doi: 10.3389/fimmu.2022.784046 35370999 PMC8965741

[49] Meyer J, Daniels SU, Grindler S, Tröscher-Mußotter J, Alaedin M, Frahm J, et al. Effects of a Dietary L-Carnitine Supplementation on Performance, Energy Metabolism and Recovery from Calving in Dairy Cows. Animals (Basel). 2020;10(2):E342. doi: 10.3390/ani10020342 32098123 PMC7070952

[50] Kraft W, Dürr UM. Klinische Labordiagnostik in der Tiermedizin. 7. Auflage ed. Stuttgart, Germany: Schattauer; 2014.

[51] Hammon HM, Steinhoff-Wagner J, Schönhusen U, Metges CC, Blum JW. Energy metabolism in the newborn farm animal with emphasis on the calf: endocrine changes and responses to milk-born and systemic hormones. Domest. Anim. Endocrinol. 2012;43(2):171–85. doi: 10.1016/j.domaniend.2012.02.005 22480719

[52] Duncan NB, Johnson PJ, Crosby MJ, Meyer AM. Serum Chemistry and Hematology Changes in Neonatal Stock-Type Foals During the First 72 Hours of Life. J. Equine Vet. Sci. 2020;84:102855. 31864462 10.1016/j.jevs.2019.102855

[53] Aoki T, Ishii M. Hematological and Biochemical Profiles in Peripartum Mares and Neonatal Foals (Heavy Draft Horse). J. Equine Vet. Sci. 2012;32(3):170–6.

[54] Ingvartsen K, Moyes K. Nutrition, immune function and health of dairy cattle. Animal: an international journal of animal bioscience. 2012;7:1–11. 23031687 10.1017/S175173111200170X

[55] Dänicke S, Kowalczyk J, Renner L, Pappritz J, Meyer U, Kramer R, et al. Effects of conjugated linoleic acids fed to dairy cows during early gestation on hematological, immunological, and metabolic characteristics of cows and their calves. J Dairy Sci. 2012;95(7):3938–53. doi: 10.3168/jds.2011-4879 22720948

[56] de Souza JS, Laureano-Melo R, Herai RH, da Conceição RR, Oliveira KC, da Silva IDCG, et al. Maternal glyphosate-based herbicide exposure alters antioxidant-related genes in the brain and serum metabolites of male rat offspring. NeuroToxicology. 2019;74:121–31. doi: 10.1016/j.neuro.2019.06.004 31226268

[57] Hammon DS, Evjen IM, Dhiman TR, Goff JP, Walters JL. Neutrophil function and energy status in Holstein cows with uterine health disorders. Vet Immunol Immunopathol. 2006;113(1–2):21–9. doi: 10.1016/j.vetimm.2006.03.022 16740320

[58] Scalia D, Lacetera N, Bernabucci U, Demeyere K, Duchateau L, Burvenich CJJoDS. In vitro effects of nonesterified fatty acids on bovine neutrophils oxidative burst and viability. J Dairy Sci. 2006;89(1):147–54. doi: 10.3168/jds.S0022-0302(06)72078-1 16357277

