# Supplementary figures and images for: Influences of glyphosate residues and different concentrate feed proportions in dairy cow rations during early gestation on performance, blood parameters, functional properties and DNA damage of blood cells in cows and their offspring
Source: PLoS One. 2023 Jun 9;18(6):e0286995. doi: 10.1371/journal.pone.0286995 (PMC10256144; doi:10.1371/journal.pone.0286995)

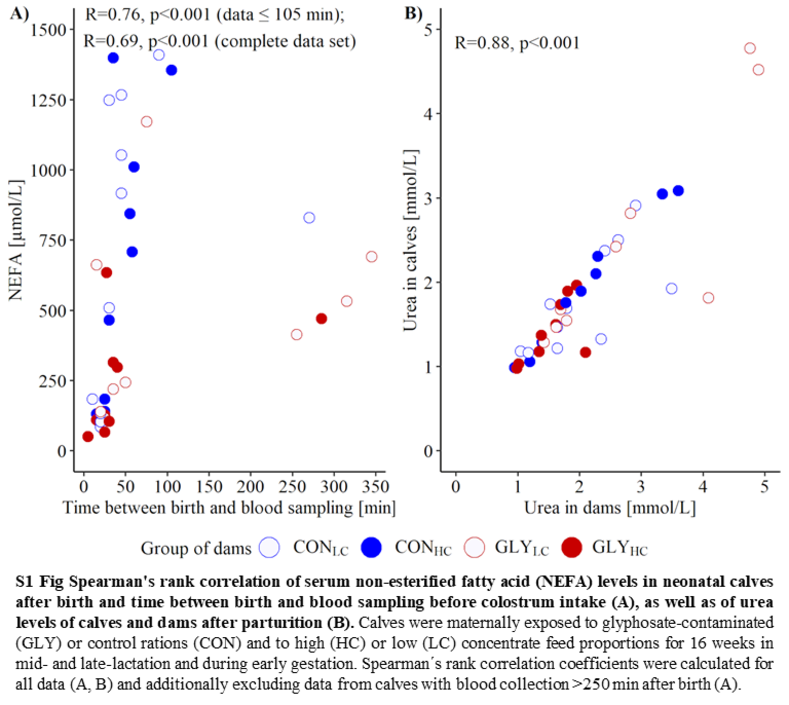

Supplement: S1 Fig — Spearman’s rank correlation of serum non-esterified fatty acid (NEFA) levels in neonatal calves after birth and time between birth and blood sampling before colostrum intake (A), as well as of urea levels of calves and dams after parturition (B). Calves were maternally exposed to glyphosate-contaminated (GLY) or control rations (CON) and to high (HC) or low (LC) concentrate feed proportions for 16 weeks in mid- and late-lactation and during early gestation. Spearman´s rank correlation coefficients were calculated for all data (A, B) and additionally excluding data from calves with blood collection >250 min after birth (A). (TIF) [file pone.0286995.s001.tif]
